# Against COVID‐19 vaccination of healthy children

**DOI:** 10.1111/bioe.13015

**Published:** 2022-03-25

**Authors:** Steven R. Kraaijeveld, Rachel Gur‐Arie, Euzebiusz Jamrozik

**Affiliations:** ^1^ Wageningen University & Research Wageningen The Netherlands; ^2^ Berman Institute of Bioethics Johns Hopkins University Baltimore Maryland USA; ^3^ Oxford‐Johns Hopkins Global Infectious Disease Ethics (GLIDE) Collaborative, Oxford, UK, and Baltimore Maryland USA; ^4^ Ethox and Wellcome Centre for Ethics and Humanities, Big Data Institute, Li Ka Shing Centre for Health Information and Discovery University of Oxford Oxford UK

**Keywords:** child vaccination, COVID‐19 vaccination, ethics, health policy, mandatory vaccination, vaccination

## Abstract

COVID‐19 vaccination of children has begun in a number of countries with provisional regulatory approval and public support. This article provides an ethical analysis of COVID‐19 vaccination of healthy children. Specifically, we present three of the strongest arguments that might justify COVID‐19 vaccination of children: (a) an argument from paternalism, (b) an argument from indirect protection and altruism, and (c) an argument from global eradication. We offer a series of objections to each of these arguments to show that none of them is currently tenable. Given the minimal direct benefit of COVID‐19 vaccination for healthy children, the potential for rare risks to outweigh these benefits and to undermine vaccine confidence, the substantial evidence that COVID‐19 vaccination confers adequate protection to risk groups whether or not healthy children are vaccinated and that current vaccines do not provide sterilizing immunity, and given that eradication of the virus is neither feasible nor a high priority for global health, we argue that routine COVID‐19 vaccination of healthy children is currently ethically unjustified. Since mandates for children have already been implemented in some places (e.g., California) and may be considered elsewhere, we also present two additional arguments explicitly against making COVID‐19 vaccination mandatory for children.

## 1 INTRODUCTION

In an increasing number of countries, COVID‐19 vaccines are being approved for use in children aged 12 to 15 and even in children as young as six months.^1^ In the United States, the Federal Drug Administration (FDA) has authorized emergency use of the Pfizer‐BioNTech COVID‐19 vaccine in children 5 through 11 years of age.^2^ The European Medicines Agency (EMA) is currently also considering extending the use of the Pfizer‐BioNTech COVID‐19 vaccine for this age group.^3^ Vaccinating children against COVID‐19 would presumably be part of a larger vaccination strategy intended to increase vaccine uptake in order to control the pandemic and reestablish normal social and economic life.^4^

This article presents an analysis of the ethics of vaccinating healthy children against COVID‐19 by responding to the strongest arguments that might favor such an approach.^5^ In particular, we present three arguments that might justify routine^6^ COVID‐19 vaccination of children, based on (a) an argument from paternalism, (b) an argument from indirect protection and altruism, and (c) an argument from the global public health aim of COVID‐19 eradication.^7^ We offer a series of objections to each respective argument to show that, given the best available data, none of them is tenable. These arguments, which might be compelling for childhood vaccination against other diseases and in different circumstances,^8^ do not appear to hold in the case of COVID‐19 with the currently available vaccines. Given the present state of affairs and all things considered, COVID‐19 vaccination of healthy children is ethically unjustified.

If one accepts our conclusion that routine vaccination of healthy children against COVID‐19 is ethically unjustified, then it follows that coercion, which is an ethically problematic issue in itself, is even less warranted. Nonetheless, mandatory vaccination of healthy children against COVID‐19 is already being considered—and, in some places, implemented—as a way of increasing vaccine uptake.^9^ We therefore also provide two objections specifically against making COVID‐19 vaccination mandatory for children, which center on additional ethical concerns about overriding the autonomy of parents and legal guardians and of children who are capable of making autonomous decisions. If vaccinating healthy children against COVID‐19 is ethically problematic, then coercing vaccination is even less acceptable—but even if vaccinating healthy children against COVID‐19 should at some future point be considered more defensible (e.g., should a much more favorable cost–benefit analysis emerge), important ethical objections against coercive mandates will still remain.

## 2 ARGUMENT FROM PATERNALISM

The first argument in favor of childhood vaccination for COVID‐19 derives from paternalistic considerations and holds that routine vaccination of healthy children is justified because it is in the best interests of the would‐be vaccinated children. The argument from paternalism suggests that COVID‐19 vaccination will, all things considered, benefit children the most (or cause them the least harm). Given that routine vaccination is the most effective way to ensure vaccine uptake, it is therefore justified for the sake of the health and well‐being of children themselves.

### 2.1 Objection 1: Low risk of COVID‐19 morbidity and mortality to children

According to the best available data, healthy children are at a much lower risk of severe illness from COVID‐19 and are less susceptible to infection than older adults.^10^ In contrast to many other vaccine‐preventable diseases, healthy children are at low risk of severe COVID‐19 infection, morbidity, and mortality.^11^ Hospitalization of children with COVID‐19 is rare, although emerging data suggest that children with severe underlying comorbidities are at higher risk.^12^ Deaths among healthy children due to COVID‐19 are very rare; for example, a large study in Germany found no deaths among children aged 5–11 without comorbidities.^13^ We agree with the assessment that COVID‐19 is not a pediatric public health emergency.^14^

Earlier concerns that the Delta variant might be associated with significantly greater morbidity and/or mortality in children do not appear to be supported by the latest data. A recent study of 258,790 children aged 5–17 years in the UK, for instance, found that illness from the Delta (B.1.617.2) variant resembled illness from the Alpha (B.1.1.7) variant, with short illness duration and similar symptom burden.^15^ Overall, the burden of COVID‐19 in children appears to be similar to or lower than that of typical seasonal influenza in the winter (unlike the much higher disease burden of COVID‐19 in adults).^16^ In 2020, 198 children aged <17 officially died of COVID‐19 in the United States.^17^ In 2021, with Delta being the predominant variant, that number increased to 378,^18^ which is comparable to the official number of children aged <17 who died in the 2018–2019 influenza season in the United States (i.e., 372).^19^ Mortality of children in Spain is low, with 8 deaths per 100,000 in children aged 0–9, and 18 deaths per 100,000 in children aged 10–19 years.^20^ In Australia, with high testing rates and local transmission of the Delta (B.1.617.2) variant, 2864 (27%) of the 10,782 cases of COVID‐19 in New South Wales were among those aged 0 to ≤18 years, and the majority of children (98%) had asymptomatic or mild infection.^21^ In the Netherlands, the official COVID‐19 death statistics published by the National Institute for Public Health and Environment (RIVM) are not broken down by age under 50, because “so few people under 50 die [that] RIVM groups together people of all ages up to and including 49,” with this group making up only 0.7% of the total number of people who died from COVID‐19 to date. It must also be noted that none of these data specify relevant comorbidities, yet most children who become severely ill or die from COVID‐19 have one or more underlying medical conditions.^22^ For infection with the Omicron variant, the severity of disease outcome was found to be significantly lower for all ages, including pediatric age groups, compared to Delta.^23^

Furthermore, post‐infection immunity has been found to be at least as effective as vaccination at protecting against disease due to reinfection with COVID‐19.^24^ An increasingly large body of evidence suggests that immunity after previous severe acute respiratory syndrome coronavirus 2 (SARS‐CoV‐2) infection is at least as robust as vaccine‐induced immunity.^25^ Childhood exposure to SARS‐CoV‐2, which, as previously discussed, is generally associated with mild viral illness, may offer protection against more severe illness in adulthood.^26^ To date, hundreds of millions of children have already been infected with COVID‐19. For children with immunity from previous infection, the potential benefits of vaccination are likely to be lower than for children without immunity; in fact, health authorities in Norway no longer recommend vaccinating children aged 12–15 who have recovered from COVID‐19.^27^ Given that the risks of the vaccines are not negligible, as we discuss in the next section, the case for vaccinating all children is therefore even less compelling when this includes large numbers of children who have already recovered from a previous SARS‐CoV‐2 infection.

It has sometimes been maintained that children often suffer significant post‐acute symptoms (also known as “long covid”) even after mild or asymptomatic infection.^28^ The preliminary data for studies supporting such an association have lacked control groups and therefore must be interpreted with caution.^29^ The idea that healthy children suffer significant post‐acute symptoms after mild or asymptomatic infection is not supported by more careful analysis of current evidence.^30^ A large‐scale recent estimate in the UK found that rates of symptoms 12–16 weeks after COVID‐19 infection in children were not statistically different from rates of symptoms among controls.^31^ Relatedly, it is biologically implausible that an infection that is usually mild or asymptomatic in children would commonly result in severe post‐infection symptoms; post‐COVID‐19 fatigue in adults was found to be strongly correlated with the severity of illness.^32^ Therefore, at this point, protecting healthy children against “long covid” does not in itself provide a strong argument for routinely vaccinating all healthy children. Should adequately controlled future data show that “long covid” more substantially affects healthy children, then this would add more weight to the argument that COVID‐19 vaccination is justified for the sake of healthy children themselves.^33^

As COVID‐19 may pose more serious risks in some children (e.g., children with obesity or severe comorbidities), vaccinating those children may be better justified by appeals to their own interests.^34^ It should be noted, however, that not all “vulnerable” groups are necessarily at increased risk of severe illness from COVID‐19. A recent study, for instance, found that immunocompromised children and young people in the UK were at no increased risks of severe COVID‐19.^35^ In any case, in light of present knowledge, it is much more difficult to justify vaccination of all children for their own sake, given the relatively low vaccine‐generated benefits and mild average disease severity.^36^ These low expected benefits need, moreover, to be balanced against potential risks, which will be addressed in the following section.

### 2.2 Objection 2: Known risks and unknown long‐term vaccine safety profile for children

The case for vaccinating healthy children against COVID‐19 for their own sake is undermined by uncertainty; that is, by the currently poorly characterized potential for rare, harmful outcomes associated with the vaccines in children. Public safety data from the Pfizer‐BioNTech clinical trials in children included 2,260 participants aged 12 to 15, of which 1,131 received the vaccine.^37^ In addition to a small sample size, the trial follow up period was of short duration; therefore, no reliable data presently exist for rare or longer‐term vaccine‐related harms.^38^ Though common adverse events occurring less than 6 months after vaccination may be ruled out, the risks of rare or delayed adverse outcomes can simply not yet be evaluated.^39^ Should vaccine harms occur, they will be revealed in the general pediatric population only after thousands or millions of children are already vaccinated, which would also risk seriously undermining vaccine confidence. The restriction of AstraZeneca vaccines to older age groups due to blood clotting events early on in the COVID‐19 vaccination rollout, as well as reports of increased rates of vaccine‐related myocarditis among younger age groups illustrates that rare risks are sometimes more common in younger age groups and might sometimes outweigh benefits in children.^40^ Severe cardiac manifestations such as myocarditis and pericarditis are now recognized as rare risks of the COVID‐19 vaccines.^41^ Myocarditis‐induced deaths following COVID‐19 vaccination have been documented in adolescents as well as in adults.^42^ The risk of vaccine‐caused myocarditis appears to be higher in younger age groups—especially males—compared to older groups.^43^ Sweden and Denmark, for instance, recently announced that they are halting use of Moderna's COVID‐19 vaccine for younger age groups after reports of rare cardiovascular side‐effects.^44^ Sweden, in fact, has decided against recommending COVID‐19 vaccines for children aged 5–11 altogether.^45^ France and Germany have also announced that they will no longer offer the Moderna vaccine to people under the age of 30 due to elevated risks of heart inflammation.^46^ The U.K. Joint Committee on Vaccination and Immunization (JCVI) has moreover recommended against vaccinating healthy children (i.e., children who do not have underlying health conditions that increase their risk from severe COVID‐19). Upon reviewing the evidence for vaccination in children aged 12–15, the JCVI concluded that for this population, “the health benefits from vaccination are marginally greater than the potential known harms.”^47^ The JCVI recommendation concerns children aged 12–15. For children aged 5–11 (the group for whom the U.S. FDA has recently authorized emergency use), the balance for vaccination is presumably less favorable, given that COVID‐19 morbidity and mortality rates decrease with younger age groups.^48^ The difference of opinion among experts and regulators suggests, at a minimum, that it is currently uncertain whether the benefits of mRNA vaccines for children outweigh the risks.^49^

Although COVID‐19 might pose more serious risks for children with severe underlying comorbidities, so that some potential vaccine risks may be more justified by potential benefits in such groups, there is reason to think that a uniform approach for all such children may be problematic. Common vaccine side effects, for instance, include fever,^50^ which for some vulnerable children may in itself pose significant risks. For the group of vulnerable children, then, who are not homogenous in terms of health status and susceptibilities, it would be preferable for COVID‐19 vaccination recommendations to be tailored at an individual level, as recommended by their pediatricians (who, after all, are arguably in the best position to provide such children with medical care). That not all vulnerable children appear to be at increased risk of severe outcomes from COVID‐19 underscores this idea.^51^

Vaccines have been recalled in the past after adverse effects in children were identified when the vaccine was already in routine use.^52^ In some cases, the adverse effects occurred many months after vaccine administration.^53^ The lack of long‐term safety data therefore warrants caution about vaccinating children against COVID‐19. Given that the combination of known vaccine risks and uncertainties (i.e., poorly characterized risks) might outweigh the limited benefits of COVID‐19 vaccination to healthy children, routine vaccination is ethically unjustified. Should positive long‐term safety data become available, this assessment might change. However, because of the low expected benefits for healthy children rooted in the best available evidence, justifying COVID‐19 vaccination by appealing to children's own interests will most likely remain ethically questionable.

In sum, vaccination of healthy children against COVID‐19 cannot presently be defended on paternalistic grounds.

## 3 ARGUMENT FROM INDIRECT PROTECTION AND ALTRUISM

The second argument is grounded in the potential benefits that vaccinating healthy children against COVID‐19 can provide to others. According to this argument, routine vaccination of children against COVID‐19 is ethically justified because healthy children should get vaccinated in order to protect vulnerable groups.

### 3.1 Objection 3: Children are not a major driver of transmission

Children are both substantially less susceptible to COVID‐19 infection and, if infected, are significantly less likely than adults to infect others.^54^ Most secondary infections directly attributable to children tend to occur within households.^55^ Yet the secondary attack rate for children to household members is low compared to adults.^56^ Since high community transmission in adults is the main driver of COVID‐19 epidemics—and infection of children—as well as disease burden, the public health benefits of vaccinating children in terms of transmission reduction (even if current vaccines were to provide sterilizing immunity, which, as we will discuss, they do not) are likely to be small and may be negligible where a high proportion of adults are already vaccinated.^57^

Moreover, if vaccination of adults and vulnerable children is maintained at a high level, as discussed in the next objection, then the public health consequences of the spread of the virus among healthy children and from them to others will be limited.

### 3.2 Objection 4: Vulnerable groups can protect themselves and current vaccines do not provide sterilizing immunity

Some vaccines (e.g., influenza) are much less effective in certain vulnerable groups (e.g., the elderly). When, in addition, non‐vulnerable groups are significant spreaders of a virus (e.g., children in the case of influenza), there may be a strong prima facie case for vaccinating the non‐vulnerable group—even if that group does not stand to benefit as much from the vaccine as the vulnerable group, for whose sake vaccine policy could in part be ethically justified.^58^ On neither count, however, does this reasoning seem to hold for COVID‐19.

For COVID‐19, vaccines are safe and effective in higher‐risk groups, including older adults and the immunocompromised,^59^ and significantly reduce the risk of severe illness even when vaccinated groups are exposed to substantial community transmission.^60^ While there are some people for whom the current COVID‐19 vaccines are contraindicated (e.g., those with severe allergies), this group appears to be small.^61^ It is therefore not the case that vulnerable groups cannot protect themselves, which would make routine vaccination of less vulnerable groups—children, in this case—more compelling. Moreover, as argued above, children are not major drivers of COVID‐19 transmission. As such, there is no strong ethical justification for COVID‐19 vaccination of healthy children for the sake of vulnerable groups.

It has been argued that people have a moral obligation to contribute to population or “herd” immunity by getting vaccinated.^62^ However, in the case of COVID‐19, it now appears unlikely that elimination via herd immunity is a possibility.^63^ Furthermore, as previously discussed, COVID‐19 vaccination is highly effective in vulnerable groups. The case for routinely vaccinating children in order that they might contribute to herd immunity is therefore weak, especially since it has become clear that the current COVID‐19 vaccines do not provide sterilizing immunity.^64^ Protection against infection with the Omicron variant falls to zero percent within a few months of the second dose of vaccine, and a similar pattern is observed for third doses.^65^ Vaccinated individuals, once infected, transmit SARS‐CoV‐2 infection to others at similar rates to unvaccinated individuals.^66^ This significantly deflates the argument for indirect protection as a justification for routine vaccination of children. However, given that transmission may still be reduced through vaccination, there may be more circumscribed instances where indirect protection weighs more strongly in favor of vaccinating children. For example, a vulnerable individual who has few contacts outside the household might receive a short term benefit if a child who lives with them is vaccinated, to the extent that this might at least temporarily reduce the chance of the child passing the virus onto them. At the same time, given the presently uncertain safety profile of COVID‐19 vaccines for children and the little direct benefit that they stand to derive from it, indirect protection arguments are still ethically questionable to the extent that they rely on children being used as a mere means for the protection of others.^67^

There are also other reasons to think that the indirect protection argument is less apt in the case of COVID‐19. Unlike in the case of vaccines for some other pathogens, very few people in risk groups will be unable to get vaccinated, provided that access is unconstrained. First, because most COVID‐19 vaccines are not live vaccines (meaning that they are safe for immunocompromised people); and, second, because there are multiple different vaccine platforms, meaning that in rare cases where someone has an allergy to a product in one particular vaccine, they may be offered an alternative one.

Even if COVID‐19 vaccines are highly effective (say 95%–99%) against severe disease, there might be a small group (say 1%–5%) in whom protection is weaker and who may therefore be better off if everyone were vaccinated. Nevertheless, since vaccination of the majority of adults will already significantly reduce the probability that those less protected by vaccination will be infected in the first place, this reasoning is hardly sufficient to justify routine COVID‐19 vaccination for all children at this point—at least not until there is a vaccine for this population with a well‐confirmed, very high safety profile.

That few people will in principle be unable to get vaccinated does not imply, of course, that everyone who is eligible will get vaccinated. That may be unwarranted optimism; vaccine uptake among adults mostly likely will not reach high levels in some places.^68^ Nevertheless, if it should be the case that vaccine uptake is not sufficiently high in adult populations, the burden appears to rest on adult populations, rather than on children.^69^

### 3.3 Objection 5: A questionable case for altruism

While children with at least some degree of decisional autonomy (e.g., teenagers) may have an obligation to take precautions against infecting others in certain cases,^70^ this obligation is significantly weakened when others are able to effectively protect themselves and when vaccines do not provide sterilizing immunity. As we have argued above, this is the case for COVID‐19. Nonetheless, one may still want to argue that, even if there is no moral obligation for children to get vaccinated against COVID‐19 for the sake of others, there should still be space for them to potentially make the altruistic choice to nevertheless get vaccinated.^71^ That is, in the absence of an obligation to get vaccinated and even if children do not stand to benefit individually from vaccination—and perhaps even in the case of a net cost to children as a group—they could still individually choose to accept the risks of vaccination for the sake of others (assuming that vaccines, while not providing sterilizing immunity, do have a significant effect on transmission). Given that one cannot be certain that by getting vaccinated against COVID‐19 one inevitably prevents harm, and if there are reasons that one does not have a strong moral obligation to get vaccinated (e.g., in the case of children), then getting vaccinated might nevertheless be seen as an altruistic act when done from the right motives.^72^ COVID‐19 vaccination of healthy children would facilitate such an altruistic choice.

While it may be a good thing to allow room for altruistic COVID‐19 vaccination decisions, this is not a sufficient justification for routine COVID‐19 vaccination of healthy children—not only because of the ethical issues surrounding routine vaccination outlined so far, but also because the group of children who might make genuine altruistic decisions is only a subset of the larger group of all children. While parents and legal guardians are de facto decision‐makers for children in early infancy, the point at which children obtain decisional autonomy is complex and may be subject to cultural differences.^73^ Aside from a widely recognized age of majority at 18 years, there is no universal age of consent for children regarding medical decisions as such, although 16 years is often recognized as the age at which some children may at least in some cases take medical decisions in the absence of parental consent.^74^ Even a 14‐year‐old “may have sufficient capacity to understand and consent” to a particular treatment, “when risks are minimal and the benefits of a proposed therapy are clear.”^75^ In the case of vaccination against COVID‐19, however, when the relevant data are still being collected and when experts are still assessing and re‐assessing the associated risks and benefits, it is implausible to think that children would be able to understand and reason well about the associated risks and benefits.

Younger children are in any case not autonomous in their ability to make medical decisions, thus relying for these decisions on their parents and legal guardians. The argument from altruism does not hold for these children, because altruism presupposes decisional autonomy; insofar as children lack autonomy, they cannot make an altruistic choice to vaccinate. At the same time, parents and guardians acting on behalf of their children cannot simply subsume the child's decision: one cannot act altruistically *through* someone else (one's child in this case). If parents wished to vaccinate their healthy children against COVID‐19 for the sake of others, then this would *not* be an altruistic choice. It would be a case of parents instrumentally using their children for the benefit of others. There are, of course, ethical reasons why parents ought not to solely regard their own children's interests as being worthy of moral consideration. Parents may have good reasons to vaccinate their child against an infectious disease even if they do not consider the risk of this disease to be substantial for their own child; for example, because their child frequently interacts with another child who is more vulnerable to the disease and cannot get vaccinated.

From a public health ethics perspective, treating children as a mere means to serve other people's or collective interests, if it can be justified, at the very least requires sufficiently large benefits to others and sufficiently small costs to children, which does not seem to be the case for COVID‐19 vaccination.^76^ Given the upshot of the discussion so far, including that vulnerable children and adults can be adequately protected by getting vaccinated and that people can still spread infection post‐vaccination, the case of COVID‐19 does not appear to raise sufficiently compelling reasons for parents to vaccinate their children solely or even primarily for the sake of others.

It must also be noted that healthy children who face very low risks from the virus have already been disproportionately harmed by non‐pharmaceutical interventions against COVID‐19, like school closures and lockdowns, all of which were primarily for the benefit of older and more vulnerable people.^77^ A great deal has been demanded of and given by these children—ought we really to ask for more?

All in all, COVID‐19 vaccination of healthy children is not justified on the grounds that healthy children should get vaccinated against COVID‐19 in order to protect others.

## 4 ARGUMENT FROM GLOBAL ERADICATION

The third argument is grounded in a pandemic “endgame” scenario: COVID‐19 vaccination of healthy children is justified because it is necessary for the global eradication of the virus. The idea is that the global reduction of SARS‐CoV‐2 incidence to zero and the ultimate cessation of vaccine programs and control measures (e.g., as in the case of smallpox) is the most ethically appropriate goal for global public health. In order to reach this goal, it is necessary to vaccinate healthy children against COVID‐19. Since no pandemic respiratory virus has ever been eradicated,^78^ this goal is in our view implausible. Yet, since several versions of this argument have appeared and may appeal to some policymakers, it is arguably worth refuting.^79^

This argument might rely on at least three claims regarding unbridled transmission of the virus, namely that ongoing transmission will: (a) lead to the evolution of viral variants that are more harmful, perhaps also for children; (b) make the virus more likely to evolve to evade vaccine‐derived immunity; and/or (c) *ceteris paribus* make the long‐term cost‐effectiveness of eradication more favorable than control. We provide objections to each of these claims in turn.

### 4.1 Objection 6: Evidence against the evolution of more harmful variants

Evolutionary fitness is primarily determined by transmissibility rather than virulence (i.e., propensity to cause harm), although these two terms are often confused or conflated.^80^ Insofar as viruses readily infect human hosts, there is an evolutionary cost to causing (fatal) harm, even if this does not exclude the possibility of viral variants becoming somewhat more harmful than their predecessors.^81^ Should variants evolve to be more harmful particularly for children, then the argument from paternalism might be strengthened (i.e., to the extent that children stand to benefit more from vaccination). However, this scenario is in our view improbable. Several seasonal coronaviruses (which also have variants) have continued to cause predominantly mild “common cold” illness in healthy children despite persistence as seasonal globally endemic viruses for decades or centuries;^82^ even SARS, caused by a far more virulent coronavirus than COVID‐19, is not particularly harmful to children.^83^ Furthermore, the virulence of SARS was one factor that made this disease relatively easy to control and eliminate (i.e., because those infected were readily identifiable) compared to COVID‐19, where mild or asymptomatic illness is far more common. The notion that more harmful variants of the virus might evolve therefore does not constitute a particularly compelling argument for routine COVID‐19 vaccination of healthy children—neither for their own sake, nor for the sake of global public health goals.

### 4.2 Objection 7: The immunity evasion argument is self‐defeating or highly costly

The notion that unbridled transmission would make the virus more likely to escape vaccine‐derived immunity makes the eradication argument either self‐defeating or incredibly costly. Aside from the fact that current vaccines do not prevent infection or transmission, if certain variants really are highly efficient at evading vaccine‐derived immunity—or, worse still, if more variants continuously evolve to evade vaccines more efficiently—then attempts at eradication through global vaccination, and the strong evolutionary selection pressures this entails, will be met with diminishing returns for the costs of such a program.

Insofar as vaccine evasion is significant, eradication would necessitate prolonged—perhaps indefinite—non‐pharmaceutical measures while up to 100% of the global population is vaccinated (including children), and/or the development of vaccines producing sterilizing immunity, including against escape variants. Given the enormous social and economic costs of prolonged non‐vaccine control measures as well as the costs of developing multiple generations of vaccines, the attractiveness of such a strategy diminishes. Moreover, both vaccine evasion and mild disease severity in children make the alternative “endgame” of global COVID‐19 endemicity (discussed below) both more plausible^84^ and arguably more ethically acceptable, especially given the many other unmet needs in global health.

### 4.3 Objection 8: One cannot assume cost‐effectiveness of eradication over control

The claim that eradication would be more cost‐effective in the long run than control remains an open question to some extent, as it does for many vaccine‐preventable diseases.^85^ Nevertheless, given that historically the efforts of global public health have successfully eradicated only one disease (smallpox), and given also that there have been significant stumbling blocks in the “last mile” of polio eradication, it appears unlikely that COVID‐19 eradication is feasible in the near term with current vaccines, due to their insufficient prevention of infection and/or transmission.^86^ The costs of such an approach have to be squared against investing in other potential global public health goals—global scourges of children such as malaria, tuberculosis, pneumococcus, diarrheal disease, and measles (as discussed below).

While a questionable end in itself, to the extent that COVID‐19 eradication would be supported by vaccinating healthy children, this goal would probably not, all things considered, be in the best interest of children. In fact, it would arguably be unethical to prioritize COVID‐19 eradication by universal vaccination of children, because there are currently far more pressing health concerns than COVID‐19 for the global population of children. The pandemic has already had a deleterious effect on routine childhood vaccine coverage, which is a serious issue that must be addressed and weighed against investing limited resources in eradicating COVID‐19.^87^ Measles, for example, which has re‐emerged during the COVID‐19 pandemic in Pakistan and the wider region, arguably poses a significantly bigger threat to children.^88^ Measles kills over 100,000 children every year for want of vaccine access—many more children than have died from COVID‐19 to date.^89^ Furthermore, the measles virus has so far demonstrated limited clinically significant immune escape in the face of vaccination,^90^ while it is widely held that measles is a candidate for an eradicable vaccine preventable disease.^91^ These conditions are not met, or remain uncertain, for COVID‐19. Even if they were met, global health policy should, insomuch as it is directly concerned with the health of children, prioritize measles eradication and many other health goals before considering universal childhood vaccination for COVID‐19.

Since low‐income countries have little incentive to participate in a COVID‐19 eradication campaign by universal vaccination while many other critical health needs of children are unmet, SARS‐CoV‐2 will inevitably become a globally endemic virus.^92^ Yet this is likely to produce very little morbidity or mortality insofar as the majority of adults and are fully vaccinated. Over time, the age at first infection will continue to fall for COVID‐19, such that, as for other coronaviruses, people will be universally infected in the early years of life and experience mild re‐infections every few years.^93^ While it is possible that vulnerable older adults will continue to face significant disease burden, as they do for other coronaviruses despite prior infection,^94^ this burden can be controlled with an appropriate use and extension of existing vaccines. If we wish to minimize harms from COVID‐19, it would be better to vaccinate vulnerable older adult populations around the world in low‐ and middle‐income countries, who stand to benefit much more from getting vaccinated and for whom access is still scarce, than children in high‐income countries.^95^ Eradication of COVID‐19 is therefore currently neither a feasible nor an ethically justifiable goal; its likely low long‐term global disease burden, once most adults are vaccinated, will soon be insufficient for prioritization above other, more pressing, global health problems.

Thus, given the objections, routine vaccination of children is not justified on the grounds that it is required to globally eradicate COVID‐19.

## 5 OBJECTIONS AGAINST MANDATES

While the ethics of vaccinating healthy children against COVID‐19 is still being debated around the world, mandatory vaccination of healthy children for COVID‐19 has already been implemented in some places, like California and Costa Rica, and may be considered elsewhere.^96^ The World Health Organization (WHO) defines a COVID‐19 vaccine mandate as a way to “compel vaccination by direct or indirect threats of imposing restrictions in cases of non‐compliance,” which can be ethically justified under certain circumstances (e.g., to protect the health and well‐being of the public), even if it interferes with individual freedom and autonomy.^97^ Some have argued that selective mandates are ethically justifiable for specific populations, such as paternalistic mandates for those who are at highest risk of severe illness from COVID‐19.^98^

However, if our ethical objections to routine vaccination of healthy children against COVID‐19 are convincing—if one accepts that routine vaccination is at least presently unjustified—then it must follow that coercion, to the extent that this would require still further ethical justification, is also unwarranted. Even among populations for which there may be more pressing reasons to increase vaccine uptake than for children, like healthcare workers, mandatory vaccination already involves serious ethical issues and may cause collateral harms.^99^

Nevertheless, given that the discussion about mandates is already underway and is likely to persist, we present two additional objections specifically against mandating vaccination of healthy children against COVID‐19, which might be considered in addition to any relevant ethical problems related to vaccine mandates in general.

### 5.1 Objection 9: Limiting parental autonomy

Mandates for children to be vaccinated against COVID‐19 would limit and, depending on their nature, even override the autonomy of parents and guardians to make decisions about the health of their children. This requires ethical justification as such, but it demands stronger justification in proportion to the level of coercion that mandates would involve.^100^ When mandates are in place, the actors who make decisions for the health and well‐being of children de facto become governments and public health officials rather than parents, although less coercive measures (e.g., small fines) might allow some parents to opt out and thereby retain decisional autonomy.^101^

To justify mandates that would limit or override parental autonomy, there needs to be at least some indication that parents and guardians might not be adequately discharging their duties to safeguard the health and well‐being of their children.^102^ Should neglect of children's basic interests be demonstrated, then there might be a legitimate reason for states to intervene and to coerce parents and guardians into making choices that are better aligned with their children's interests and well‐being.^103^

In the case of COVID‐19, however, there is no such indication. There is no compelling reason to assume that by not vaccinating their healthy children against COVID‐19, parents are failing in their duty to uphold their children's best interests. As previous objections have shown, it is currently questionable whether the balance of benefits and risks even weighs in favor of vaccinating healthy children against COVID‐19. Not vaccinating one's children against COVID‐19 therefore does not presently constitute a clear case of parental failure, which makes the coercion of parental vaccination decisions for one's children unwarranted on those grounds.

If one accepts our conclusions that vaccinating healthy children against COVID‐19 is not required in order to protect others and not necessary for the public health goal of eradicating COVID‐19, then it follows that other‐regarding and public health considerations also do not justify making COVID‐19 vaccination mandatory for children.

### 5.2 Objection 10: Mandates preclude altruism for autonomous children

As previously discussed, perhaps some healthy children (e.g., teenagers) can autonomously make the altruistic choice to get vaccinated for others. Clearly, overriding the autonomy of these children through coercive measures will be as ethically problematic as for parents and legal guardians. However, there is an additional element to consider.

Encouraging children with decisional autonomy to get vaccinated for the sake of others may be a good thing, insofar as the vaccines are safe for them in the long term and insofar as children are able to adequately understand the associated risks and benefits.^104^ Yet altruism crucially requires freedom; it depends on the proper kind of self‐chosen motive to act for the sake of someone else.^105^ Thus, even if some healthy children might choose to get vaccinated for the sake of others, mandates would preclude the possibility of freely acting on laudable altruistic motives. This argument is important for any attempt to enforce civic duties (e.g., through payments or fines), because regulating and especially enforcing other‐regarding behavior arguably undermines solidarity, trust, reciprocity, and other communal values.^106^ The same argument also affects potential altruistic behavior in the case of coercive vaccination policies for adults.^107^ For healthy children who can make their own decisions, mandating vaccination against COVID‐19 would undercut the altruistic motives that these children might otherwise heed.

## 6 CONCLUSION

We have presented three of the most compelling arguments that might justify routine vaccination of healthy children against COVID‐19: an argument from paternalism or the best interests of children, an argument from indirect protection or the best interests of vulnerable others, and an argument from global eradication or the best interests of a global COVID‐19 public health endgame. Through sustained objections to each respective argument, we have shown that, given the present evidence regarding the disease and the available vaccines, none is ultimately sufficient to justify routine COVID‐19 vaccination of healthy children. We also elaborated two further objections specifically against mandating COVID‐19 vaccination for children: one based on ethical issues surrounding coercion and parental autonomy, and the other based on the idea that mandates would undermine potentially altruistic decisions of autonomous children to get vaccinated for the sake of others. All things considered, neither routine nor mandatory vaccination of healthy children against COVID‐19 is currently ethically justified.

## CONFLICT OF INTEREST

The authors declare no conflict of interest.

## Footnotes

1 Mandavilli, A. (2021, May 10). F.D.A. authorizes Pfizer‐BioNTech vaccine for children 12 to 15. *The New York Times*. https://www.nytimes.com/2021/05/10/health/pfizer-vaccine-children-kids.html; CNA. (2021, June 5). *Sinovac's COVID‐19 vaccine gains China approval for emergency use in children, adolescents*. https://www.channelnewsasia.com/news/asia/covid-19-sinovac-vaccine-china-approval-children-14952682; Swiss Agency for Therapeutic Products. (2021, June 4). *Pfizer/BioNTech COVID‐19 vaccine approved for young people in Switzerland*. https://www.swissmedic.ch/swissmedic/en/home/news/coronavirus-covid-19/covid-19-impfstoff-pfizer-biontech-fuer-jugendliche.html; Pfizer. (2021, December 17). *Pfizer and BioNTech Provide Update on Ongoing Studies of COVID‐19 Vaccine* [Press release]. https://www.pfizer.com/news/press-release/press-release-detail/pfizer-and-biontech-provide-update-ongoing-studies-covid-19

2 U.S. Food & Drug Administration. (2021, October 29). *FDA authorizes Pfizer‐BioNTech COVID‐19 vaccine for emergency use in children 5 through 11 years of age* [Press release]. https://www.fda.gov/news-events/press-announcements/fda-authorizes-pfizer-biontech-covid-19-vaccine-emergency-use-children-5-through-11-years-age

3 European Medicines Agency. (2021, October 18). *EMA starts evaluating use of COVID‐19 vaccine Comirnaty in children aged 5 to 11*. EMA. https://www.ema.europa.eu/en/news/ema-starts-evaluating-use-covid-19-vaccine-comirnaty-children-aged-5-11

4 Gostin, L. O., Salmon, D. A., & Larson, H. J. (2021). Mandating COVID‐19 vaccines. *Journal of the American Medical Association, 325*(6), 532–533; Eberhardt, C. S., & Siegrist, C.‐A. (2020). Is there a role for childhood vaccination against COVID‐19? *Pediatric Allergy and Immunology, 32*, 9–16.

5 We focus our analysis on healthy children because, as will be discussed later in the article, there may be more compelling reasons to vaccinate at least some vulnerable children against COVID‐19. Throughout the article, where we refer simply to children, this should be taken to imply healthy children.

6 By “routine” vaccination we mean vaccination that is recommended for everyone, so that routine vaccination of children should be understood as the general recommendation that all children get vaccinated against COVID‐19, whether or not the rationale for the recommendation is emergency use. In the United States, for example, given the emergency use authorization of the Pfizer‐BioNTech COVID‐19 vaccine in children aged 5–11, routine vaccination for this age group means offering the vaccine to all eligible children (whether it is simply offered, actively encouraged, required, or even mandated).

7 We consider these three arguments to be the most ethically relevant and potentially convincing for vaccinating children against COVID‐19. However, should additional arguments for vaccinating children be raised, then these would have to be critically appraised together with the three arguments presented in this paper for an all‐things‐considered judgment. In other words, those who would argue in favor of vaccinating children against COVID‐19 would have to show that the objections against the three arguments in this paper do not hold, or that some additional argument(s) are of greater ethical significance than the arguments considered here. Thanks to an anonymous reviewer for pressing us to clarify this point.

8 Kraaijeveld, S. R. (2020). Vaccinating for whom? Distinguishing between self‐protective, paternalistic, altruistic and indirect vaccination. *Public Health Ethics, 13*(2), 190–200. https://doi.org/10.1093/phe/phaa005

9 Plotkin, S. A., & Levy, O. (2021). Considering mandatory vaccination of children for COVID‐19. *Pediatrics Perspectives*, *147*(6), e2021050531; Savulescu, J., Giubilini, A., & Danchin, M. (2021). Global ethical considerations regarding mandatory vaccination. *The Journal of Pediatrics, 231*, 10–16; BBC. (2021, 5 November). *Covid vaccine to be mandatory for children in Costa Rica*. BBC News. https://www.bbc.co.uk/news/world-latin-america-59162510; Gutman, R. (2021, November 5). COVID‐vaccine mandates for kids are coming. But are they a good idea? *The Atlantic*. https://www.theatlantic.com/health/archive/2021/11/kids-school-covid-vaccine-mandate/620622/

10 Verity, R., Okell, L. C., Dorigatti, I., Winskill, P., Whittaker, C., Imai, N., Cuomo‐Dannenburg, G., Thompson, H., Walker, P. G. T., Fu, H., Dighe, A., Griffin, J. T., Baguelin, M., Bhatia, S., Boonyasiri, A., Cori, A., Cucunubá, Z., FitzJohn, R., Gaythorpe, K., … Ferguson, N. M. (2020). Estimates of the severity of coronavirus disease 2019: A model‐based analysis. *The Lancet Infectious Diseases, 20*(6), 669–677; Bhopal, S. S., Bagaria, J., Olabi, B., & Bhopal, R. (2021). Children and young people remain at low risk of COVID‐19 mortality. *The Lancet Child & Adolescent Health, 5*(5), e12–e13.

11 Piroth, L., Cottenet, J., Mariet, A.‐S., Bonniaud, P., Blot, M., Tubert‐Bitter, P., & Quantin, C. (2021). Comparison of the characteristics, morbidity, and mortality of COVID‐19 and seasonal influenza: A nationwide, population‐based retrospective cohort study. *The Lancet Respiratory Medicine, 9*(3), 251–259.

12 Salje, H., Tran Kiem, C., Lefranq, N., Courtejoie, N., Bosetti, P., Paireau, J., Andronico, A., Hozé, N., Richet, J., Dubost, C.‐L., Le Strat, Y., Lessler, J., Levy‐Bruhl, D., Fontanet, A., Opatowsk, L., Boelle, P.‐Y., & Cauchemez, S. (2020). Estimating the burden of SARS‐CoV‐2 in France. *Science, 369*(6500), 208–211.

13 Makary, M. (2021, June 10). *Think twice before giving the COVID vax to healthy kids*. MedPage Today. http://medpagetoday.com/opinion/marty-makary/93029?vpass=1; Ledford, H. (2021). Deaths from COVID ‘incredibly rare’ among children. *Nature, 595*, 639. https://doi.org/10.1038/d41586-021-01897-w; Sorg, A., Hufnagel, M., Doenhardt, M., Diffloth, N., Schroten, H., v. Kries, R., Berner, R., & Armann, J. (2021). Risk of Hospitalization, severe disease, and mortality due to COVID‐19 and PIMS‐TS in children with SARS‐CoV‐2 infection in Germany. *MedRxiv*. https://doi.org/10.1101/2021.11.30.21267048

14 Pegden, W., Prasad, V., & Baral, S. (2021, May 19). Covid vaccines for children should not get emergency use authorization. *The BMJ Opinion*. https://blogs.bmj.com/bmj/2021/05/07/covid-vaccines-for-children-should-not-get-emergency-use-authorization

15 Molteni, E., Sudre, C. H., Canas, L. S., Bhopal, S. S., Hughes, R. C., Chen, L., Deng, J., Murray, B., Kerfoot, E., Antonelli, M., Graham, M., Kläser, K., May, A., Hu, C., Pujol, J. C., Wolf, J., Hammers, A., Spector, T. D., Ourselin, S., … Duncan, E. L. (2021). Illness characteristics of COVID‐19 in children infected with the SARS‐CoV‐2 Delta variant. *medRxiv*. https://doi.org/10.1101/2021.10.06.21264467

16 Ibid.

17 National Center for Health Statistics. (2021, November 3). *Provisional death counts for coronavirus disease 2019 (COVID‐19)*. Centers for Disease Control and Prevention. https://www.cdc.gov/nchs/nvss/vsrr/covid_weekly/index.htm

18 Ibid. It must be noted that this dataset includes deaths up to November 2021; the total number for 2021 is likely to be higher.

19 National Center for Immunization and Respiratory Diseases (NCIRD). (2021, September 29). *Estimated flu‐related illnesses, medical visits, hospitalizations, and deaths in the United States – 2018–2019 flu season*. Centers for Disease Control and Prevention. https://www.cdc.gov/flu/about/burden/2018-2019.html

20 Tagarro, A., García‐Salido, A., Martínez, E., Vega‐Piris, L., & Mellado, M. J. (2021). Low COVID‐19 mortality in Spanish children. *The Lancet Child & Adolescent Health, 5*(6), E24–E25. https://doi.org/10.1016/S2352-4642(21)00125-5

21 National Centre for Immunisation and Research and Surveillance (NCIRS). (2021). *COVID‐19 Delta variant in schools and early childhood education and care services in NSW, Australia: 16 June to 31 July 2021*. https://ncirs.org.au/covid-19-delta-variant-schools-and-early-childhood-education-and-care-services-nsw-australia-16

22 McCormick, D. W., Richardson, L. C., Young, P. R., Viens, L. J., Gould, C. V., Kimball, A., Pindyck, T., Rosenblum, H. R., Siegel, D. A., Vu, Q. M., Komatsu, K., Venkat, H., Openshaw, J. J., Kawasaki, B., Siniscalchi, A. J., Gumke, M., Leapley, A., Tobin‐D'Angelo, M., Kauerauf, J., … Koumans, E. H. on behalf of the Pediatric Mortality Investigation Team. (2021). Deaths in children and adolescents associated with COVID‐19 and MIS‐C in the United States. *Pediatrics, 148*(5), e2021052273. https://doi.org/10.1542/peds.2021-052273; Tsankov, B. K., Allaire, J. M., Irvine, M. A., Lopez, A. A., Sauvé, Vallance, B. A., & Jacobson, K. (2021). Severe COVID‐19 infection and pediatric comorbidities: A systematic review and meta‐analysis. *International Journal of Infectious Diseases, 103*, 246–256. https://doi.org/10.1016/2Fj.ijid.2020.11.163

23 Wang, L., Berger, N. A., Kaelber, N. A., Davis, P. B., Volkow, N. D., & Xu, R. (2022). Comparison of outcomes from COVID infection in pediatric and adult patients before and after the emergence of Omicron. *medRxiv*. https://doi.org/10.1101/2F2021.12.30.21268495

24 Pritchard, E., Matthews, P. C., Stoesser, N., Eyre, D. W., Gethings, O., Vihta, K.‐D., Jones, J., House, T., VanSteenHouse, H., Bell, I., Bell, J. I., Newton, J. N., Farrar, J., Diamond, I., Rourke, E., Studley, R., Crook, D., Peto, T., Walker, A. S., & Pouwels, K. B. (2021). Impact of vaccination on SARS‐CoV‐2 cases in the community: A population‐based study using the UK's COVID‐19 infection survey. *medRxiv*. https://doi.org/10.1101/2021.04.22.21255913; Gazit, S., Shlezinger, R., Perez, G., Lotan, R., Peretz, A., Ben‐Tov, A., Cohen, D., Muhsen, K., Chodick, G., & Patalon, T. (2021). Comparing SARS‐CoV‐2 natural immunity to vaccine‐induced immunity: Reinfections versus breakthrough infections. *medRxiv*. https://doi.org/10.1101/2021.08.24.21262415

25 Gazit et al., op. cit. note 24; Pilz, S., Chakeri, A., Ioannidis, J. P. A., Richter, L., Theiler‐Schwertz, V., Trummer, C., Krause, R., & Allerberger, F. (2021). SARS‐CoV‐2 re‐infection risk in Austria. *European Journal of Clinical Investigation, 51*(4), e13520. https://doi.org/10.1111/eci.13520; Neidleman, J., Luo, X., McGregor, M., Xie, G., Murray, V., Greene, W. C., Lee, S. A., & Roan, N. R. (2021). mRNA vaccine‐induced T cells respond identically to SARS‐CoV‐2 variants of concern but differ in longevity and homing properties depending on prior infection status. *eLife, 10*, e72619. https://doi.org/10.7554/eLife.72619; Turner, J. S., Kim, W., Kalaidina, E., Goss, C. W., Rauseo, A. M., Schmitz, A. J., Hansen, L., Haile, A., Klebert, M. K., Pusic, I., O'Halloran, J. A., Presti, R. M., & Ellebedy, A. H. (2021). SARS‐CoV‐2 infection induces long‐lived bone marrow plasma cells in humans. *Nature, 595*, 421–425; Haveri, A., Ekström, N., Virta, C., Österlund, P., Isosaari, E., Nohynek, H., Palmu, A. A., & Melin, M. (2021). Persistence of neutralizing antibodies a year after SARS‐CoV‐2 infection in humans. *European Journal of Immunology, 51*(12), 3202–3213. https://doi.org/10.1002/eji.202149535; Letizia, A. G., Yongchao, G., Vangeti, S., Goforth, C., Weir, D. L., Kuzmina, N. A., Balinsky, C. A., Chen, H. W., Ewing, D., Soares‐Schanoski, A., George, M.‐C., Graham, W. D., Jones, F., Bharaj, P., Lizewski, R. A., Lizewski, S. E., Marayag, J., Marjanovic, N., Miller, C. M., … Sealfon, S. C. (2021). SARS‐CoV‐2 seropositivity and subsequent infection risk in healthy young adults: A prospective cohort study. *Respiratory Medicine, 9*(7), 712–720. https://doi.org/10.1016/S2213-2600(21)00158-2; Wadman, M. (2021). SARS‐CoV‐2 infection confers greater immunity than shots. *Science, 373*(6559),1067‐1068; Pilz, S., Theiler‐Schwetz, V., Trummer, C., Krause, R., & Ioannidis, P. A. (2022). SARS‐CoV‐2 reinfections: Overview of efficacy and duration of natural and hybrid immunity. *SSRN*. https://doi.org/10.2139/ssrn.4005459

26 Rees, E. M., Waterlow, N. R., Lowe, R., & Kucharski, A. J. (2021). Estimating the duration of seropositivity of human seasonal coronaviruses using seroprevalence studies [version 3; peer review: 3 approved].*Wellcome Open Research, 6*, 138. https://doi.org/10.12688/wellcomeopenres.16701.3; Pilz et al.2022, op. cit. note 25.

27 Norwegian Institute of Public Health (NIPH). (2021, November 10). *Coronavirus vaccine – Information for the public*. https://www.fhi.no/en/id/vaccines/coronavirus-immunisation-programme/coronavirus-vaccine/#vaccination-of-children-and-adolescents

28 Buonsenso, D., Munblit, D., De Rose, C., Sinatti, D., Ricchiuto, A., Carfi, A., & Valentini, P. (2021). Preliminary evidence on long COVID in children. *Acta Paediatrica, 110*(7), 2208–2211. https://doi.org/10.1101/2021.01.23.21250375; Ludvigsson, J. F. (2020). Case report and systematic review suggest that children may experience similar long‐term effects to adults after clinical COVID‐19. *Acta Paediatrica, 110*(3), 914–921.

29 Akst, J. (2021, March 4). Kids may suffer from long COVID, but data are scarce. *TheScientist*. https://www.the-scientist.com/news-opinion/kids-may-suffer-from-long-covid-but-data-are-scarce-68511

30 Bhopal, S. S., & Absoud, M. (2021). Vaccinating children to prevent long covid? More caution is needed in interpreting current epidemiological data. *BMJ, 372*(n520), 1.

31 Ayoubkhani, D., Pawelek, P., & Gaughan, C. (2021, September 16). *Technical article: Updated estimates of the prevalence of post‐acute symptoms among people with coronavirus (COVID‐19) in the UK: 26 April 2020 to 1 August 2021*. Office for National Statistics. https://www.ons.gov.uk/peoplepopulationandcommunity/healthandsocialcare/conditionsanddiseases/articles/technicalarticleupdatedestimatesoftheprevalenceofpostacutesymptomsamongpeoplewithcoronaviruscovid19intheuk/26april2020to1august2021

32 Crameri, G. A. G., Bielecki, M., Zust, R., Buehrer, T. W., Stanga, Z., & Deuel, J. W. (2020). Reduced maximal aerobic capacity after COVID‐19 in young adult recruits, Switzerland, May 2020. *Eurosurveillance, 25*(36), 1–4.

33 Given that there are both risks and uncertainties related to COVID‐19 vaccines for children, this gives us reason to be cautious. On the other hand, uncertainty about long‐term effects of COVID‐19 on children may provide a reason to consider vaccination after all. However, given that the effects of long covid appear to be minimal, and given the already known rare but potentially severe harms of the vaccines, the balance of ethical considerations appears to be against routine vaccination of healthy children. Thanks to an anonymous reviewer for pressing us on this point.

34 It might turn out that tailored vaccination against COVID‐19 for vulnerable children will be more expensive to implement than universal vaccination. A cost–benefit analysis of this kind is important to consider when it comes to the ethics of COVID‐19 vaccination of children. At present, however, potential implementation costs provide relatively little weight against a tailored approach given that several risk factors (such as obesity) are now well described, and provided that (under a tailored approach) vaccines remain easily accessible for parents who wish to access them for their children. Thanks to an anonymous reviewer for raising this point.

35 Chappell, H., Patel, R., Driessens, C., Tarr, A. W., Irving, W. L., Tighe, P. J., Jackson, H. J., Harvey‐Cowlishaw, T., Mills, L., Shaunak, M., Gbesemete, D., Leahy, A., Lucas, J. S., Faust, S. N., & de Graaf, H. (2022). Immunocompromised children and young people are at no increased risk of severe COVID‐19. *Journal of Infection, 84*(1), 31–39.

36 We do not wish to give the impression that we reject paternalistic reasons for routine childhood vaccination altogether. Paternalism—the best interests of children—is probably the single most important justification for childhood vaccination. Even for rare diseases (e.g., polio), routine vaccination may be justified on the grounds that, while the disease is rare, the protection provided by herd immunity is in the best interest of children as a group, given the severity of the disease for children should they become infected (i.e., should herd immunity wane). As we have argued, however, these conditions do not hold for COVID‐19 in the case of children. Thanks to an anonymous reviewer for pressing us to clarify this point.

37 U.S. Food and Drug Administration. (2021, May 10). *Coronavirus (COVID‐19) update: FDA authorizes Pfizer‐BioNTech COVID‐19 vaccine for emergency use in adolescents in another important action in fight against pandemic* [Press release]. https://www.fda.gov/news-events/press-announcements/coronavirus-covid-19-update-fda-authorizes-pfizer-biontech-covid-19-vaccine-emergency-use

38 Pfizer. (2021, May 19). *About our landmark trial*. https://www.pfizer.com/science/coronavirus/vaccine/about-our-landmark-trial

39 Pegden et al., op. cit. note 14; Benn, C. S. (2021). Should we delay covid‐19 vaccination in children? *BMJ, 374*, n1687. https://doi.org/10.1136/bmj.n1687

40 Marshall, M., Ferguson, I. D., Lewis, P., Jaggi, P., Gagliardo, C., Collins, J. S., Shaughnessy, R., Caron, R., Fuss, C., Corbin, K. J. E., Emuren, L., Faherty, E., Hall, E. K., Di Pentima, C., Oster, M. E., Paintsil, E., Siddiqui, S., Timchak, D. M., & Guzman‐Cottrill, J. A. (2021). Symptomatic acute myocarditis in seven adolescents following Pfizer‐BioNTech COVID‐19 vaccination. *Pediatrics, 148*(3), e2021052478. https://doi.org/10.1542/peds.2021-052478; Vogel, G., & Couzin‐Frankel, J. (2021). Israel reports link between rare cases of heart inflammation and COVID‐19 vaccination in young men. *Science*. https://doi.org/10.1126/science.abj7796

41 Witberg, G., Barda, N., Hoss, S., Richter, I., Wiessman, M., Aviv, Y., Grinberg, T., Auster, O., Dagan, N., Balicer, R. D., & Kornowski, R. (2021). Myocarditis after Covid‐19 vaccination in a large health care organization. *New England Journal of Medicine, 385*(23), 2132–2139. https://doi.org/10.1056/NEJMoa2110737; Kim, H. W., Jenista, E. R., Wendell, D. C., Azevedo, C. F., Campbell, M. J., Darty, S. N., Parker, M. A., & Kim, R. J. (2021). Patients with acute myocarditis following mRNA COVID‐19 vaccination. *JAMA Cardiology, 6*(10), 1196–1201; Paul, J., Charles, P., Richaud, C., Caussin, C., & Diakov, C. (2021). Myocarditis revealing COVID‐19 infection in a young patient. *European Heart Journal – Cardiovascular Imaging, 21*(7), 776; Diaz, G. A., Parsons, G. T., Gering, S. K., Meier, A. R., Hutchinson, I. V., & Robicsek, A. (2021). Myocarditis and pericarditis after vaccination for COVID‐19. *JAMA, 326*(12), 1210–1212; Simone, A., Herald, J., Chen, A., Gulati, N., Shen, A. Y., Lewin, B., & Lee, M. (2021). Acute myocarditis following COVID‐19 mRNA vaccination in adults aged 18 years or older. *JAMA Internal Medicine, 181*(12), 1668–1670. https://doi.org/10.1001/jamainternmed.2021.5511

42 Gill, J. R., Tashjian, R., & Duncanson, E. (2022). Autopsy histopathologic cardiac findings in two adolescents following the second COVID‐19 vaccine dose. *Archives of Pathology & Laboratory Medicine*. https://doi.org/10.5858/arpa.2021-0435-SA; Choi, S., Lee, S., Seo, J., Kim, M., Jeon, Y. H., Park, J. H., Lee, J. K., & Yeo, N. S. (2021). Myocarditis‐induced sudden death after BNT162b2 mRNA COVID‐19 vaccination in Korea: Case report focusing on histopathological findings. *Journal of Korean Medical Science, 36*(40), e286. https://doi.org/10.3346/jkms.2021.36.e286; Mevorach, D., Anis, E., Cedar, N., Bromberg, M., Haas, E. J., Nadir, E., Olsha‐Castell, S., Arad, D., Hasin, T., Levi, N., Asleh, R., & Amir, O. (2021). Myocarditis after BNT162b2 mRNA vaccine against Covid‐19 in Israel. *New England Journal of Medicine, 385*, 2140–2149. https://doi.org/10.1056/NEJMoa2109730

43 National Center for Immunization and Respiratory Diseases (NCIRD). (2021, September 8). *Myocarditis and pericarditis after mRNA COVID‐19 vaccination*. Centers for Disease Control and Prevention. https://www.cdc.gov/coronavirus/2019-ncov/vaccines/safety/myocarditis.html; Hause, A. M., Baggs, J., Marquez, P., Myers, T. R., Gee, J., Su, J. R., Zhang, B., Thompson, D., Shimabukuro, T. T., & Shay, D. K. (2021). COVID‐19 vaccine safety in children aged 5–11 years—United States, November 3–December 19, 2021. *Centers for Disease Control and Prevention Morbidity and Mortality Weekly Report, 70*(51–52), 1755–760; Mevorach et al., op. cit. note 43; Høeg, T. B., Krug, A., & Mandrola, J. (2021). SARS‐CoV‐2 mRNA vaccination‐associated myocarditis in children ages 12‐17: A stratified national database analysis. *medRxiv*. https://doi.org/10.1101/2021.08.30.21262866; for a criticism of the preceding study, see Munro, C. (2021). Covid‐19: Study that claimed boys are at increased risk of myocarditis after vaccination is deeply flawed, say critics. *BMJ*, *374*, n2251.

44 Ahlander, J., & Jacobsen, S. (2021). *Sweden, Denmark pause Moderna COVID‐19 vaccine for younger age groups*. Reuters. https://www.reuters.com/business/healthcare-pharmaceuticals/sweden-pauses-use-moderna-covid-vaccine-cites-rare-side-effects-2021-10-06/

45 Ahlander, J. (2022). *Sweden decides against recommending COVID vaccines for kids aged 5‐11*. Reuters. https://www.reuters.com/world/europe/sweden-decides-against-recommending-covid-vaccines-kids-aged-5-12-2022-01-27

46 Hart, R. (2021, November 10). Germany, France restrict Moderna's covid vaccine for under‐30s over rare heart risk—Despite surging cases. *Forbes*. https://www.forbes.com/sites/roberthart/2021/11/10/germany-france-restrict-modernas-covid-vaccine-for-under-30s-over-rare-heart-risk-despite-surging-cases/

47 Public Health England*. (2021, September 3).JCVI issues updated advice on COVID‐19 vaccination of children aged 12 to 15* [Press release]. https://www.gov.uk/government/news/jcvi-issues-updated-advice-on-covid-19-vaccination-of-children-aged-12-to-15

48 Twohig, K. A., Nyberg, T., Zaidi, A., Thelwall, S., Sinnathamby, M. A., Aliabadi, S., Seaman, S. R., Harris, R. J., Hope, R., Lopez‐Bernal, J., Gallagher, E., Charlett, A., De Angelis, D., Presanis, A. M., & Dabrera, G. (2022). Hospital admission and emergency care attendance risk for SARS‐CoV‐2 delta (B.1.617.2) compared with alpha (B.1.1.7) variants of concern: a cohort study. *The Lancet Infectious Diseases, 22*, 35. https://doi.org/10.1016/S1473-3099(21)00475-8

49 For an overview of different positions by governments around the world regarding vaccinating children against COVID‐19, see Fidler, S., Forero, J., & Lieber, D. (2021, 13 November). As the U.S. races to vaccinate kids against Covid‐19, some countries hold back. *Wall Street Journal*. https://www.wsj.com/articles/as-the-u-s-races-to-vaccinate-kids-against-covid-19-some-countries-hold-back-11636799400; see also Lavine, J. S. (2021). Vaccinating children against SARS‐CoV‐2. *BMJ, 373*, n1197. https://doi.org/10.1136/bmj.n1197; Benn, C. S. (2021). Should we delay covid‐19 vaccination in children? *BMJ, 374*, n1687. https://doi.org/10.1136/bmj.n1687

50 World Health Organization. (2021, February 19). *Coronavirus disease (COVID‐19): Vaccines safety*. https://www.who.int/news-room/q-a-detail/coronavirus-disease-(covid-19)-vaccines-safety

51 Chappell et al., op. cit. note 36.

52 Collignon, P., Doshi, P., & Jefferson, T. (2010). Ramifications of adverse events in children in Australia. *BMJ, 340*, c2994. https://doi.org/10.1136/bmj.c2994

53 Jamrozik, E., Heriot, G., Bull, S., & Parker, M. (2021). Vaccine‐enhanced disease: case studies and ethical implications for research and public health. *Wellcome Open Research, 6*, 154; Stowe, J., Andrews, N., Gringas, P., Quinnell, T., Zaiwalla, Z., Shneerson, J., & Miller, E. (2020). Reassessment of the risk of narcolepsy in children in England 8 years after receipt of the AS03‐adjuvanted H1N1 pandemic vaccine: A case‐coverage study. *PLoS Medicine, 17*(9), e1003225. https://doi.org/10.1371/journal.pmed.1003225

54 Monod, M., Blenkinsop, A., Xi, X., Hebert, D., Bershan, S., Tietze, S., Baguelin, M., Bradley, V. C., Chen, Y., Coupland, H., Filippi, S., Ish‐Horowicz, J., McManus, M., Mellan, T., Gandy, A., Hutchinson, M., Unwin, H. J. T., van Elsland, S., Vollmer, M. A. C., … Ratmann, O. (2021). Age groups that sustain resurging COVID‐19 epidemics in the United States. *Science, 371*(6536), eabe8372. https://doi.org/10.1126/science.abe8372

55 Lei, H., Xu, X., Xiao, S., Wu, X., & Shu, Y. (2020). Household transmission of COVID‐19—A systematic review and meta‐analysis. *Journal of Infection, 81*(6), 993–994.

56 Kim, J., Choe, Y. J., Lee, J., Park, Y. J., Park, O., Han, M. S., Kim, J.‐H., & Choi, E. H. (2021). Role of children in household transmission of COVID‐19. *Archives of Disease in Childhood, 106*, 709–711; for an earlier metanalysis with similar findings, see Madewell, Z. J., Yang, Y., Longini, I. M. Jr., Halloran, M. E., & Dean, N. E. (2021). Household transmission of SARS‐CoV‐2: A systematic review and meta‐analysis. *JAMA Network Open, 3*(12), e2031756; for an earlier network prospective study in Spain, see Soriano‐Arandes, A., Gatell, A., Serrano, P., Biosca, M., Campillo, F., Capdevila, R., Fàbrega, A., Lobato, Z., López, N., Moreno, A. M., Poblet, M., Riera‐Bosch, M. T., Rius, N., Ruiz, M., Sánchez, A., Valldepérez, C., Vilà, M., Pineda, V., Lazcano, U., … Soler‐Palacín, P. (2021). Household severe acute respiratory syndrome Coronavirus 2 transmission and children: A network prospective study. *Clinical Infectious Diseases*, 73(6), e1261–e1269.

57 Lee, A., & Bhopil, S. (2021, February 2). Coronavirus school closures: What's the evidence? *The Conversation*. https://theconversation.com/coronavirus-school-closures-whats-the-evidence-154210

58 Bambery, B., Selgelid, M., Maslen, H., Pollard, A. J., & Savulescu, J. (2013). The case for mandatory flu vaccination of children. *The American Journal of Bioethics, 13*(9), 38–40; Bambery, B., Douglas, T., Selgelid, M. J., Maslen, H., Giubilini, A., Pollard, A. J., & Savulescu, J. (2018). Influenza vaccination strategies should target children. *Public Health Ethics, 11*(2), 221–234.

59 Dagan, N., Barda, N., Kepten, E., Miron, O., Perchik, S., Katz, M. A., Hernán, M. A., Lipsitch, M., Reis, B., & Balicer, R. D. (2021). BNT162b2 mRNA Covid‐19 vaccine in a nationwide mass vaccination setting. *New England Journal of Medicine, 384*(15), 1412–1423.

60 Menni, C., Klaser, K., May, A., Polidori, L., Capdevila, J., Louca, P., Sudre, C. H., Nguyen, L. H., Drew, D. A., Merino, J., Hu, C., Selvachandran, S., Antonelli, M., Murray, B., Canas, L. S., Molteni, E., Graham, M. S., Modat, M., Joshi, A. D., … Spector, T. D. (2021). Vaccine side‐effects and SARS‐CoV‐2 infection after vaccination in users of the COVID Symptom Study app in the UK: A prospective observational study. *The Lancet Infectious Diseases, 21*(7), 939–949. https://doi.org/10.1016/S1473-3099(21)00224-3

61 Rasmussen, T. H., Mortz, C. G., Georgsen, T. K., Rasmussen, H. M., Kjaer, H. F., & Bindslev‐Jensen, C. (2021). Patients with suspected allergic reactions to COVID‐19 vaccines can be safely revaccinated after diagnostic work‐up. *Clinical and Translational Allergy, 1*(5), E12044. https://doi.org/10.1002/clt2.12044

62 Giubilini, A., Douglas, T., & Savulescu, J. (2018). The moral obligation to be vaccinated: Utilitarianism, contractualism, and collective easy rescue. *Medicine, Health Care and Philosophy, 21*, 547–560.

63 Aschwanden, C. (2021). Five reasons why COVID herd immunity is probably impossible. *Nature, 591*, 520–522.

64 Vashishtha, V. M., & Kumar, P. (2022). Looking to the future: Is a universal coronavirus vaccine feasible? *Expert Review of Vaccines, 30*, 1–4. https://doi.org/10.1080/14760584.2022.2020107; Singanayagam, A., Hakki, S., Dunning, J., Madon, K. J., Crone, M. A., Koycheva, A., Derqui‐Fernandez, N., Barnett, J. K., Whitfield, M. G., Varro, R., Charlett, A., Kundu, R., Fenn, J., Cutajar, J., Quinn, V., Conibear, E., Barclay, W., Freemont, P. S., Taylor, G. P., … Lalvani, A. (2021). Community transmission and viral load kinetics of the SARS‐CoV‐2 delta (B.1.617.2) variant in vaccinated and unvaccinated individuals in the UK: A prospective, longitudinal, cohort study. *The Lancet Infectious Diseases, 22*(2), 183–195. https://doi.org/10.1016/S1473-3099(21)00648-4; Federman, J. (2022, January 18). *Israel study: 4th vaccine shows limited results with omicron*. ABC News. https://abcnews.go.com/Health/wireStory/israel-study-4th-vaccine-shows-limited-results-omicron-82312196

65 UK Health Security Agency. (2022, December 23). COVID‐19 vaccine surveillance report. Week 42. GOV.UK. https://www.gov.uk/government/publications/ukhsa-investigation-of-novel-sars-cov-2-variants-of-concern-england-technical-briefing-33-23-december-2021

66 Wilder‐Smith, A. (2021). What is the vaccine effect on reducing transmission in the context of the SARS‐CoV‐2 delta variant? The Lancet Infectious Diseases 21, S1473‐3099. https://doi.org/10.1016/S1473-3099(21)00690-3

67 Giubilini, A. (2021, September 13). The double ethical mistake of vaccinating children against COVID‐19. *Practical Ethics Blog*. https://blog.practicalethics.ox.ac.uk/2021/09/the-double-ethical-mistake-of-vaccinating-children-against-covid-19/#more-15400; see also Malm, H., & Navin, M. (2020). Pox parties for grannies? Chickenpox, exogenous boosting, and harmful injustices. *The American Journal of Bioethics, 20*(9), 45–57. https://doi.org/10.1080/15265161.2020.1795528

68 De Figueiredo, A., Simas, C., Karafillakis, E., Patersen, P., & Larson, H. J. (2020). Mapping global trends in vaccine confidence and investigating barriers to vaccine uptake: A large‐scale retrospective temporal modelling study. *The Lancet, 396*(10255), 898–908.

69 cf. Ibid.

70 Verweij, M. (2005). Obligatory precautions against infection. *Bioethics, 19*(4), 323–335.

71 Kraaijeveld, op. cit. note 8.

72 cf. the discussion of altruism in Kraaijeveld, S. R. (2020). COVID‐19: Against a lockdown approach. *Asian Bioethics Review, 13*(2), 195–212. https://doi.org/10.1007/s41649-020-00154-y

73 Helwig, C. (2006). The development of personal autonomy throughout cultures. *Cognitive Development, 21*(4), 458–473.

74 Coughlin, K. W. (2018). Medical decision‐making in paediatrics: Infancy to adolescence. *Paediatrics & Child Health, 23*(2), 138–146.

75 Ibid: 139.

76 Giubilini, A., Gupta, S., & Heneghan, C. (2021). A focused protection vaccination strategy: Why we should not target children with COVID‐19 vaccination policies. *Journal of Medical Ethics, 47*(8), 565–566; Bhopal, S. S., Bagaria, J., & Olabi, B. (2021). Children and young people remain at low risk of COVID‐19 mortality. *Lancet Child Adolescent Health, 5*(5), e12–e13. https://doi.org/10.1016/S2352-4642(21)00066-3

77 Von Bismarck‐Osten, C., Borusyak, K., & Schönberg, U. (2020). *The role of schools in transmission of the SARS‐CoV‐2 virus: Quasi‐experimental evidence from Germany* (Ruhr Economic Papers 882). https://doi.org/10.4419/96973021; UNICEF. (2021). *Life in lockdown: Child and adolescent mental health and well‐being in the time of COVID‐19*. UNICEF Office of Research – Innocenti. https://www.unicef-irc.org/publications/pdf/Life-in-Lockdown.pdf

78 Heriot, G. S., & Jamrozik, E. (2021). Imagination and remembrance: What role should historical epidemiology play in a world bewitched by mathematical modelling of COVID‐19 and other epidemics? *History and Philosophy of the Life Sciences, 43*, 81. https://doi.org/10.1007/s40656-021-00422-6

79 Wilson, N., Mansoor, O. D., Boyd, M. J., Kvalsvig, A., & Baker, M. G. (2021). We should not dismiss the possibility of eradicating COVID‐19: Comparisons with smallpox and polio. *BMJ Global Health, 6*, e006810. https://doi.org/10.1136/bmjgh-2021-006810; a number of policy‐makers and leaders have suggested some variation of this idea, whether or not explicitly as a policy of global or local “eradication.” In Australia, “zero covid” is still official policy at the time of writing (see Scott, J. (2021, October 1). *Australia pushes forward with plan to end covid‐zero isolation*. Bloomberg. https://www.bloomberg.com/news/articles/2021-10-01/australia-pushes-forward-with-plan-to-end-covid-zero-isolation).

80 Van Dorp, L., Richard, D., Tan, C. C. S., Shaw, L. P., Acman, M., & Balloux, F. (2020). No evidence for increased transmissibility from recurrent mutations in SARS‐CoV‐2. *Nature Communications, 11*, 5986.

81 Alexander, M. (1981). Why microbial predators and parasites do not eliminate their prey and hosts. *Annual Reviews of Microbiology, 35*, 113–133; Kirchner, J. W., & Roy, B. A. (2002). Evolutionary implications of host–pathogen specificity: Fitness consequences of pathogen virulence traits. *Evolutionary Ecology Research, 4*, 27–48.

82 Fehr, A. R., & Perlman, S. (2015). Coronaviruses: An overview of their replication and pathogenesis. *Methods in Molecular Biology, 1282*, 1–23.

83 Leung, T. F., Wong, G. W. K., Hon, K. L. E., & Fok, T. F. (2003). Severe acute respiratory syndrome (SARS) in children: Epidemiology, presentation and management. *Paediatric Respiratory Reviews, 4*(4), 334–339.

84 Lavine, J. S., Bjornstad, O. N., & Antia, R. (2021). Immunological characteristics govern the transition of COVID‐19 to endemicity. *Science*, 371(6530), 741–745. https://doi.org/10.1126/science.abe6522; Heriot & Jamrozik, op. cit. note 78.

85 Bart, K. J., Foulds, J., & Patriarca, P. (1996). Global eradication of poliomyelitis: Benefit‐cost analysis. *Bulletin of the World Health Organization, 74*(1), 35–45; Sicuri, E., Evans, D. B., & Tediosi, F. (2015). Can economic analysis contribute to disease elimination and eradication? A systematic review. *PLoS One, 10*(6), e0130603. https://doi.org/10.1371/journal.pone.0130603

86 Lavine et al., op. cit. note 84.

87 Eberhardt, C. S., & Siegrist, C.‐A. (2020). Is there a role for childhood vaccination against COVID‐19? *Pediatric Allergy and Immunology, 32*, 9–16.

88 Rana, M. S., Alam, M. M., Ikram, A., Salman, M., Mere, M. O., Usman, M., Umair, M., Zaidi, S. S. Z., & Arshad, Y. (2021). Emergence of measles during the COVID‐19 pandemic threatens Pakistan's children and the wider region. *Nature Medicine, 27*, 1127–1128. https://doi.org/10.1038/s41591-021-01430-6

89 World Health Organization. (2020). *Worldwide measles deaths climb 50% from 2016 to 2019 claiming over 207 500 lives in 2019*. https://www.who.int/news/item/12-11-2020-worldwide-measles-deaths-climb-50-from-2016-to-2019-claiming-over-207-500-lives-in-2019

90 Yang, L., Grenfell, B. T., & Mina, M. J. (2019). Measles vaccine immune escape: Should we be concerned? *European Journal of Epidemiology, 34*, 893–896. https://doi.org/10.1007/s10654-019-00574-7

91 Moss, W. J., & Strebel, P. (2011). Biological feasibility of measles eradication. *The Journal of Infectious Diseases, 204*(Suppl. 1), S47–S53.

92 Philips, N. (2021). The coronavirus is here to stay —Here's what that means. *Nature, 590*, 382–384. https://doi.org/10.1038/d41586-021-00396-2; Veldhoen, M., & Simas, J. P. (2021). Endemic SARS‐CoV‐2 will maintain post‐pandemic immunity. *Nature Reviews Immunology, 21*, 131–132. https://doi.org/10.1038/s41577-020-00493-9

93 Edridge, A. W. D., Kaczorowska, J., Hoste, A. C. R., Bakker, M., Klein, M., Loens, K., Jebbink, M. F., Matser, A., Kinsella, C. M., Rueda, P., Ieven, M., Goossens, H., Prins, M., Sastre, P., Deijs, M., & Van der Hoek, L. (2020). Seasonal coronavirus protective immunity is short‐lasting. *Nature Medicine, 26*, 1691–1693; see also Rees, E. M., Waterlow, N. R., Centre for the Mathematical Modelling of Infectious Diseases COVID‐19 Working Group, Lowe, R., & Kucharski, A. J. (2021). Estimating the duration of seropositivity of human seasonal coronaviruses using seroprevalence studies. *Wellcome Open Research, 6*, 138. https://doi.org/10.12688/wellcomeopenres.16701.1; Lavine et al., op. cit. note 84.

94 Patrick, D. M., Petric, M., Skowronski, D. M., Guasparini, R., Booth, T. F., Krajden, M., McGeer, P., Bastien, N., Gustafson, L., Dubord, J., Macdonald, D., David, S. T., Srour, L. F., Parker, R., Andonov, A., Isaac‐Renton, J., Loewen, N., McNabb, G., McNabb, A., … Brunham, R. C. (2006). An outbreak of human coronavirus OC43 infection and serological cross‐reactivity with SARS coronavirus. *Canadian Journal of Infectious Diseases and Medical Microbiology, 17*(6), 330–336.

95 Gur‐Arie, R., Kraaijeveld, S. R., & Jamrozik, E. (2021). An ethical analysis of vaccinating children against COVID‐19: Benefits, risks, and issues of global health equity. *Wellcome Open Research, 6*(252). https://doi.org/10.12688/wellcomeopenres.17234.2

96 Office of Governor Gavin Newsom. (2021, October 1). *California becomes first state in nation to announce COVID‐19 vaccine requirements for schools*. https://www.gov.ca.gov/2021/10/01/california-becomes-first-state-in-nation-to-announce-covid-19-vaccine-requirements-for-schools/; BBC, op. cit. note 9; Plotkin & Levy, op. cit. note 9; Savulescu et al., op. cit. note 9.

97 World Health Organization Ethics and COVID‐19 Working Group. (2021). *COVID‐19 and mandatory vaccination: Ethical considerations and caveats*, p. 1. https://www.who.int/publications/i/item/WHO-2019-nCoV-Policy-brief-Mandatory-vaccination-2021.1

98 Williams, B. M. (2021). The ethics of selective mandatory vaccination for COVID‐19. *Public Health Ethics*, https://doi.org/10.1093/phe/phab028

99 Gur‐Arie, R., Jamrozik, E., & Kingori, P. (2021). No jab, no job? Ethical issues in mandatory COVID‐19 vaccination of healthcare personnel. *BMJ Global Health, 6*, e004877. https://doi.org/10.1136/bmjgh-2020-004877; Waters, A. (2022). Covid‐19: Reassess effects of mandatory vaccination on staffing, urge unions. *The BMJ, 376*, o139. https://doi.org/10.1136/bmj.o139

100 Giubilini, A. (2019). *The ethics of vaccination*. Palgrave Macmillan.

101 It should be noted here that one ethical worry with mandates is that it may create an unfair situation where some parents and guardians may be able to “afford” (i.e., literally when mandates take the form of a fine) to disregard mandates and to retain decisional autonomy in disregarding COVID‐19 vaccination for their children, while others (e.g., less affluent parents and guardians) are significantly less able and thus less free to opt out.

102 Pierik, R. (2018). Mandatory vaccination: An unqualified defence. *Journal of Applied Philosophy, 32*(2), 381–398.

103 Pierik, R. (2020). Vaccination policies: Between best and basic interests of the child, between precaution and proportionality. *Public Health Ethics, 13*(2), 201–214.

104 Here, again, it should be noted that getting vaccinated for the sake of others is only a possibility when a vaccine significantly reduces one's chances of transmitting infection to others.

105 Seglow, J. (2004). Altruism and freedom. In J. Seglow (Ed.), *The ethics of altruism* (pp. 145–171). Frank Cass Publishers; Kraaijeveld, S. R. (2020). COVID‐19: Against a lockdown approach. *Asian Bioethics Review, 13*(2), 195–212. https://doi.org/10.1007/s41649-020-00154-y

106 Savulescu, J. (2020). Good reasons to vaccinate: Mandatory or payment for risk? *Journal of Medical Ethics, 47*(2), 78–85.

107 cf. Kraaijeveld, op. cit. note 107.

